# Matching novel face and voice identity using static and dynamic facial images

**DOI:** 10.3758/s13414-015-1045-8

**Published:** 2016-01-05

**Authors:** Harriet M. J. Smith, Andrew K. Dunn, Thom Baguley, Paula C. Stacey

**Affiliations:** Nottingham Trent University, Nottingham, UK; Psychology Division, Nottingham Trent University, Burton Street, Nottingham, NG1 4BU UK

**Keywords:** Static, Dynamic, Face, Voice, Crossmodal matching

## Abstract

Research investigating whether faces and voices share common source identity information has offered contradictory results. Accurate face–voice matching is consistently above chance when the facial stimuli are dynamic, but not when the facial stimuli are static. We tested whether procedural differences might help to account for the previous inconsistencies. In Experiment 1, participants completed a sequential two-alternative forced choice matching task. They either heard a voice and then saw two faces or saw a face and then heard two voices. Face–voice matching was above chance when the facial stimuli were dynamic and articulating, but not when they were static. In Experiment 2, we tested whether matching was more accurate when faces and voices were presented simultaneously. The participants saw two face–voice combinations, presented one after the other. They had to decide which combination was the same identity. As in Experiment 1, only dynamic face–voice matching was above chance. In Experiment 3, participants heard a voice and then saw two static faces presented simultaneously. With this procedure, static face–voice matching was above chance. The overall results, analyzed using multilevel modeling, showed that voices and dynamic articulating faces, as well as voices and static faces, share concordant source identity information. It seems, therefore, that above-chance static face–voice matching is sensitive to the experimental procedure employed. In addition, the inconsistencies in previous research might depend on the specific stimulus sets used; our multilevel modeling analyses show that some people look and sound more similar than others.

Redundant information offered by faces and voices facilitates everyday social communication (Campanella & Belin, 2007). Testing whether novel (and therefore unfamiliar) faces and voices can be accurately matched provides a measure of the extent to which faces and voices offer redundant source identity information. Although some research has suggested that crossmodal matching of novel faces and voices is only possible when dynamic visual information about articulatory patterns is available (Kamachi, Hill, Lander, & Vatikiotis-Bateson, 2003; Lachs & Pisoni, 2004a), other research has suggested that it is possible to match static faces to voices because they offer concordant source identity information (Krauss, Freyberg, & Morsella, 2002; Mavica & Barenholtz, 2013; Smith, Dunn, Baguley, & Stacey, 2015). We tested whether differences between the experimental procedures across previous studies might account for these apparently inconsistent results.

## A crucial role for dynamic visual articulatory patterns?

Idiosyncratic speaking styles dictate what voices sound like and how faces move (Lander, Hill, Kamachi, & Vatikiotis-Bateson, 2007; Yehia, Rubin, & Vatikiotis-Bateson, 1998). Audiovisual speech perception researchers have emphasized the existence of links between auditory and visual sensory modalities (e.g., Kuhl & Meltzoff, 1984; MacDonald & McGurk, 1978; McGurk & MacDonald, 1976) and have demonstrated that participants can match sequentially presented dynamic images of articulating faces to speakers (Lachs & Pisoni, 2004a), even when the voice and face are producing different sentences (Kamachi et al., 2003; Lander et al., 2007). The conclusion that crossmodal source identity information is contingent on encoding dynamic visual articulatory patterns has been supported by studies finding that static face–voice matching performance is at chance level (Kamachi et al., 2003; Lachs & Pisoni, 2004a). The importance of time-varying articulatory information is underlined by the fact that participants can match faces and voices using movement information alone. Studies isolating articulatory movement using a point-light technique have produced accurate matching of utterances to dynamic displays (Lachs & Pisoni, 2004b; Rosenblum, Smith, Nichols, Hale, & Lee, 2006).

Other research challenges the conclusion that dynamic visual information is crucial to crossmodal matching. Krauss et al. (2002) showed that people could match a voice to one of two full-length static images of different people with above-chance accuracy. Whereas the studies observing chance-level matching performance using static faces and voices used stimuli of a similar age, gender, and ethnicity in each trial (e.g., Kamachi et al., 2003), Krauss et al.’s stimuli were from a wider age range (20–60 years). The stimuli were also full-length images rather than images of faces, which may have provided additional cues to inform accurate matching. However, Mavica and Barenholtz (2013) replicated Krauss et al.’s results using static headshots of age-matched stimuli, and face–voice matching was above chance in both of the experiments they reported. Similarly, Smith et al. (2015) also observed above-chance static face–voice matching. These three studies offer growing evidence that the source identity information available in static faces overlaps with the information offered by voices.

## Concordant information in faces and voices

In light of research investigating the extent to which faces and voices offer similar information about personal characteristics, above-chance static face–voice matching makes intuitive sense. Studies testing the concordance between ratings of attractiveness from static faces and voices suggest that both validly signal genetic quality (Collins & Missing, 2003; Feinberg et al., 2005; Saxton, Caryl, & Roberts, 2006; T. Wells, Baguley, Sergeant, & Dunn, 2013). Hormone levels are reflected in both faces (Penton-Voak & Chen, 2004; Perrett et al., 1998; Thornhill & Grammer, 1999) and voices (Abitbol, Abitbol, & Abitbol, 1999; Beckford, Rood, & Schaid, 1985; O’Connor, Re, & Feinberg, 2011; Pisanski, Mishra, & Rendall, 2012). A man who sounds masculine should therefore also tend to look masculine, and similarly, feminine-sounding women should tend to look feminine. In a recent study, Smith et al. (2015) asked participants to complete a number of rating scales for faces and corresponding voices. Faces and voices were presented in two separate blocks. The results showed that independent judgments about femininity and masculinity made from faces and voices were strongly and positively correlated. Positive correlations were also found between face and voice ratings of age, health, height, and weight (Smith et al., 2015). Interestingly, the strength of correlations did not vary according to whether the faces were static or dynamic. These results suggest that static face–voice matching is possible (Krauss et al., 2002; Mavica & Barenholtz, 2013; Smith et al., 2015) because faces do not need to be dynamic in order to share concordant information with voices.

## Procedural differences between studies

Procedural differences between studies may account for some of the apparently contradictory results outlined above. Audiovisual speech perception studies (e.g., Kamachi et al., 2003; Lachs & Pisoni, 2004a, b; Lander et al., 2007), have tended to use a “crossmodal matching task” (Lachs, 1999). This is a sequential two-alternative forced choice (2AFC) procedure. In the visual to auditory (V–A) condition, a face is shown and then two voices are presented at test, one after the other. In the auditory to visual (A–V) condition, this procedure is reversed: Participants hear a voice and then see two sequentially presented faces at test. At test, one of the alternatives is therefore always the same-identity target, whereas the other is a different-identity distractor. The participant must decide which of the two alternatives matches the identity of the other-modality stimulus. Studies that have used this procedure have generally emphasized the importance of dynamic articulatory information in facilitating face–voice matching; above-chance face–voice matching is typically found for dynamic but not for static faces (Kamachi et al., 2003; Lachs & Pisoni, 2004a, b; Lander et al., 2007). In contrast, the majority of experiments observing above-chance levels of matching accuracy using static facial stimuli have not used this exact procedure, making it unwise to directly compare the results. For instance, Krauss et al. (2002) presented a voice followed by two simultaneously presented full-length images. Smith et al. (2015) used a same–different procedure in which participants saw a face and heard a voice, and then had to decide whether or not the face and voice shared the same identity. Mavica and Barenholtz’s (2013) stimuli (one voice and two test faces) were presented simultaneously in Experiment 1. However, it is important to note that Mavica and Barenholtz’s second experiment replicated above-chance-level matching with static facial stimuli using the A–V condition of the standard crossmodal matching task (Lachs, 1999). Although the V–A condition was not included, this result hints that even if procedural differences across studies hold some explanatory value, additional factors may also affect performance and help to explain the existing contradictions. Nevertheless, the impact of procedural differences on face–voice matching accuracy deserves further attention.

A possible explanation for the differences in face–voice matching between static and dynamic stimuli is associated with memory demands. Some research has suggested that memory for dynamic facial images is better than that for static facial images (e.g., Christie & Bruce, 1998; Knappmeyer, Thornton, & Bülthoff, 2003; Lander & Chuang, 2005). In a review, O’Toole, Roark, and Abdi (2002) put forward two explanations for this increased memorability. According to the “representation enhancement hypothesis,” dynamic images facilitate the perception of 3-D facial structure. In the “supplemental information hypothesis,” motion is thought to provide additional signature information about the given person. Therefore, when stimuli are presented sequentially (as in a crossmodal matching task), poorer memory for static images could make it harder for participants to hold the face in working memory long enough to compare with the voice for source identity information. In an attempt to rule out memory explanations for the results of their first experiment, which detected above-chance static face–voice matching, Mavica and Barenholtz (2013) used sequential presentation in their Experiment 2. Their results did not entirely rule out an explanation for the discrepancies across studies based on memory effects. In neither experiment did Mavica and Barenholtz include a dynamic face–voice matching condition. If memory load affects performance, we might expect to find a position effect in a 2AFC task, whereby accuracy is higher if the correct other-modality stimulus appears in Position 1 rather than Position 2. Previous studies have not included analyses of responses by position, and thus the impact of this factor is unknown, although position effects for 2AFC tasks are well-documented in the literature (García-Pérez & Alcalá-Quintana, 2011; Yeshurun, Carrasco, & Maloney, 2008).

Failure to include both static and dynamic face conditions therefore prevents a direct comparison of crossmodal matching explanations based on static facial information (e.g., Krauss et al., 2002; Mavica & Barenholtz, 2013) with those focusing on dynamic facial information (e.g., Kamachi et al., 2003; Lachs & Pisoni, 2004a, b; Lander et al., 2007; Rosenblum et al., 2006). To date, only one study has directly compared matching performance using static and dynamic facial stimuli in the same experiment, and it found no difference in matching accuracy across conditions (Smith et al., 2015). Further clarification of these results using a crossmodal matching procedure will be necessary. However, as has been suggested by other results (Kamachi et al., 2003; Lachs & Pisoni, 2004a), it is feasible that participants tested using dynamic facial stimuli may significantly outperform those in static conditions because dynamic stimuli make both temporal and spatial information available to inform matching decisions.

## Aims

In the face of these contradictory results, in the experiments presented here we aimed to clarify whether static face–voice matching is possible using stimuli of the same age, sex, and ethnicity. In an attempt to tease apart the relative contributions of static and dynamic face information in facilitating crossmodal matching, performance using static and dynamic faces was compared in both Experiments 1 and 2. In case better memory for dynamic facial stimuli affects matching accuracy, memory load was varied across the experiments: In Experiment 1, all stimuli were presented sequentially, so memory load was higher, whereas in Experiment 2, face–voice combinations were presented simultaneously. In a further test of whether static face–voice matching is sensitive to procedural differences, for Experiment 3 we adopted the procedure of Krauss et al. (2002), in which the alternatives in a 2AFC task are presented simultaneously. To clarify how memory load and task type affect the results, in all three experiments we also investigated whether accuracy is higher when the correct, matching other-modality stimulus appears in Position 1 rather than Position 2.

## Experiment 1

In Experiment 1 we used a standard crossmodal matching task (Lachs, 1999) to compare static and dynamic face–voice matching. In most experiments in which this procedure has been used, the results have shown only dynamic face–voice matching to be above chance level (Kamachi et al. 2003; Lachs & Pisoni, 2004a; Lander et al., 2007; cf. Mavica & Barenholtz, 2013, Exp. 2). Informed by the balance of evidence, we expected static face–voice matching to be at chance level.

### Method

#### Design

Experiment 1 employed a 2 × 2 × 2 mixed factorial design. The between-subjects factor was Facial Stimulus Type (static or dynamic), and the within-subjects factors were Order (visual then auditory [V–A] or auditory then visual [A–V]) and Position (1 or 2). The dependent variable was matching accuracy.

#### Participants

The participants (*N =* 82) were recruited from the Nottingham Trent University Psychology Division’s Research Participation Scheme by convenience sampling. A total of 26 male and 56 female participants took part (age range *=* 18 to 66 years, *M =* 23.70, *SD =* 8.56). All participants reported having normal or corrected vision and hearing. In line with course requirements, student participants received three research credits. Ethical approval for this and subsequent experiments was granted by the university’s BLSS (Business, Law, and Social Science) College Research Ethics Committee.

#### Apparatus and materials

The stimuli were taken from the GRID audiovisual sentence corpus (Cooke, Barker, Cunningham, & Shao, 2006). The corpus features head and shoulder videos of British adults recorded against a plain background saying six-word sentences in an emotionally neutral manner. Each sentence follows the same structure: (1) command, (2) color, (3) preposition, (4) letter, (5) digit, and (6) adverb—for example, *Place red at F2 please*. A total of 18 speakers were selected from the corpus: nine male and nine female. All of the speakers were between 18 and 30 years of age and were white British with an English accent.

The stimuli were presented on an Acer Aspire laptop (screen size *=* 15.6 in., resolution *=* 1,366 × 768 pixels, Dolby Advanced Audio), with brightness set to the maximum level. The experiment ran on PsychoPy version 1.77.01 (Peirce, 2009), an open-source software package for running experiments in Python. The study used the same static faces, dynamic faces, and voices as Smith et al. (2015). Three .mpeg-format videos were randomly selected from the GRID corpus for each of the 18 speakers. The videos were selected using an online research randomizer (Urbaniak & Plous, 2013). One of the three videos was used to create static pictures of faces (.png format). The static picture for each talker was the first frame of the video. Another of the three video files was used to construct the dynamic stimuli by muting the sound. Facial stimuli measured 384 × 288 pixels and were presented for 2 s, in color. Voice recordings were also played for 2 s. To reduce background noise, participants listened to the recordings binaurally through Apple EarPods at a comfortable listening volume (30 % of the maximum). Apple EarPods have a frequency range of 5 to 21000 Hz. This is wider than the normal range of human hearing (Feinberg et al. 2005.

Four versions of the experiment were created, so that trials could be constructed using different combinations of stimuli. Each version consisted of 12 trials in total, and each trial featured three stimuli. In the V–A condition, a face (Stimulus 1) was followed by two sequentially presented voices (Stimuli 2 and 3): a target and a distractor. In the A–V condition, a voice (Stimulus 1) was followed by sequentially presented target and distractor faces (Stimuli 2 and 3). Across versions, whether someone’s face/voice appeared as Stimulus 1, 2, or 3, and whether it was used in a V–A or A–V trial, was randomly varied. The position of the same-identity other-modality stimulus at test (Position 1 or 2) was also randomly and equally varied. None of the faces or voices appeared more than once in each experimental version. Each of the four versions was used for the between-subjects manipulation of facial stimuli (static or dynamic), so in total there were eight versions of the experiment.

#### Procedure

The participants were randomly allocated to one of the eight versions of the experiment using an online research randomizer (Urbaniak & Plous, 2013). In the dynamic facial stimulus condition, participants were accurately informed that the face and the voice were saying different sentences, to prevent the use of speech-reading (Kamachi et al. 2003.

**Fig. 1:**
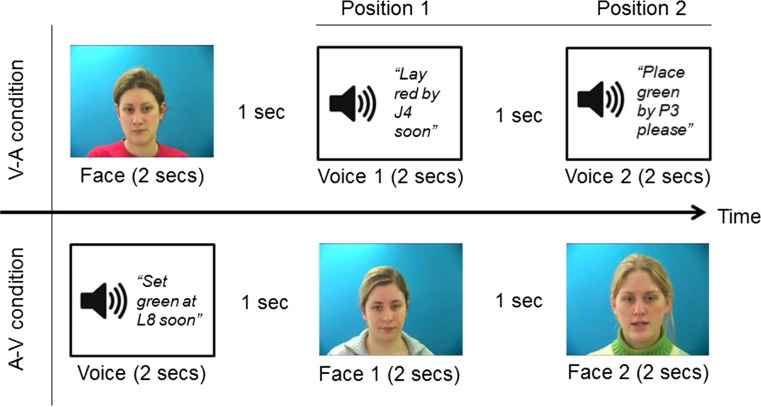
The procedure used in Experiment 1

The participants completed two counterbalanced experimental blocks. The procedure is illustrated in Fig. 1. First, participants received a practice trial, followed by six randomly ordered trials. In one block of trials, participants saw a face first. After a 1-s gap, they heard the first voice. The text “Voice 1” was visible in the middle of the screen while the recording was playing. After another 1-s gap, they heard the second voice, with the text “Voice 2” visible in the middle of the screen. In the other block of trials, participants heard a voice first, and then saw two faces, presented one after the other. Gaps of 1 s were inserted between all stimuli, and the text “Face 1” or “Face 2” appeared below each picture. At test, participants were asked to select either “1” or “2” as the face/voice that had the same identity as the first stimulus.

#### Data analysis and multilevel modeling

All data were analyzed using multilevel models so that both participants and stimuli could be treated as random effects. The random effects were fully crossed; every participant encountered all 36 stimuli (18 faces, 18 voices) in each version of the experiment. Multilevel modeling avoids aggregating data (see Smith et al. 2015; Wells et al. 2013) and inflating the risk of Type I error (Baguley, 2012; Clark, 1973; Judd, Westfall, & Kenny, 2012). Accordingly, multilevel modeling was the most appropriate analysis, because it takes into account the variability associated with individual performance and different stimuli. The variance associated with stimuli may be particularly important when investigating face–voice matching. Mavica and Barenholtz (2013) reported that matching performance varied between 35 % and 70 % for the 64 models whose faces and voices they used as stimuli. Disregarding this source of variance would risk the ecological fallacy (see Robinson, 1950), by falsely assuming that the observed patterns for participant means also occur at the level of individual trials.

### Results

Matching accuracy was analyzed using multilevel logistic regression with the lme4, version 1.06, package in R (Bates, Maechler, Bolker, & Walker, 2014). This is the same method of analysis used in Smith et al. (2015). Four nested models were compared, all fitted using restricted maximum likelihood, and with accuracy (0 or 1) as the dependent variable. The first model included a single intercept; the second included the main effects of each factor (Order, Position, and Facial Stimulus Type). The third added the two-way interactions, and the final model included the three-way interaction. This method of analysis allowed us to test for individual effects in a way similar to traditional analysis of variance (ANOVA). However, as *F* tests derived from multilevel models tend not to be accurate, we report the likelihood ratio tests provided by lme4. These are more robust and are obtained by dropping each effect in turn from the appropriate model (e.g., testing the three-way interaction by dropping it from the model including all effects, and testing the two-way interactions by dropping each effect in turn from the two-way model).

Table 1 shows the likelihood chi-square statistic (*G*^2^) and *p* value associated with dropping each effect. Table 1 also reports the coefficients and standard errors (on a log odds scale) for each effect in the full three-way interaction model. Variability for the first stimulus in each trial (the voice in the A–V condition, and the face in the V–A condition) was modeled separately from the foil stimulus. The random effect for the first stimuli captures the variability of both faces and voices, because corresponding faces and voices are highly correlated. For foils we modeled separate random effects for faces and voices, because the corresponding voice or face was never present. In the three-way model, the estimated *SD* of the first-stimulus random effect was .535; for the voice foils it was .634; and for face foils it was .484. The estimated *SD* for the participant effect was less than .0001. A similar pattern held for the null model. Thus, although individual differences were negligible in this instance, a conventional by-participants analysis that did not simultaneously incorporate the variance associated with the stimuli could be extremely misleading.

**Table 1:** Parameter estimates (*b*) and likelihood ratio tests for the 2 × 2 × 2 factorial analysis, Experiment 1: Sequential face–voice presentation

*Source*	*df*	*b*	*SE*	*G* ^*2*^	*p*
Intercept	1	0.444	0.315	–	–
Position	1	0.062	0.374	5.92	.015
Order	1	0.333	0.371	0.68	.410
Facial Stimulus Type	1	0.676	0.277	3.42	.064
Position × Order	1	0.870	0.516	0.35	.553
Position × Facial Stimulus Type	1	0.625	0.390	0.02	.884
Order × Facial Stimulus Type	1	0.775	0.382	0.59	.441
Position × Order × Facial Stimulus Type	1	1.159	0.549	4.34	.037

**Fig. 2:**
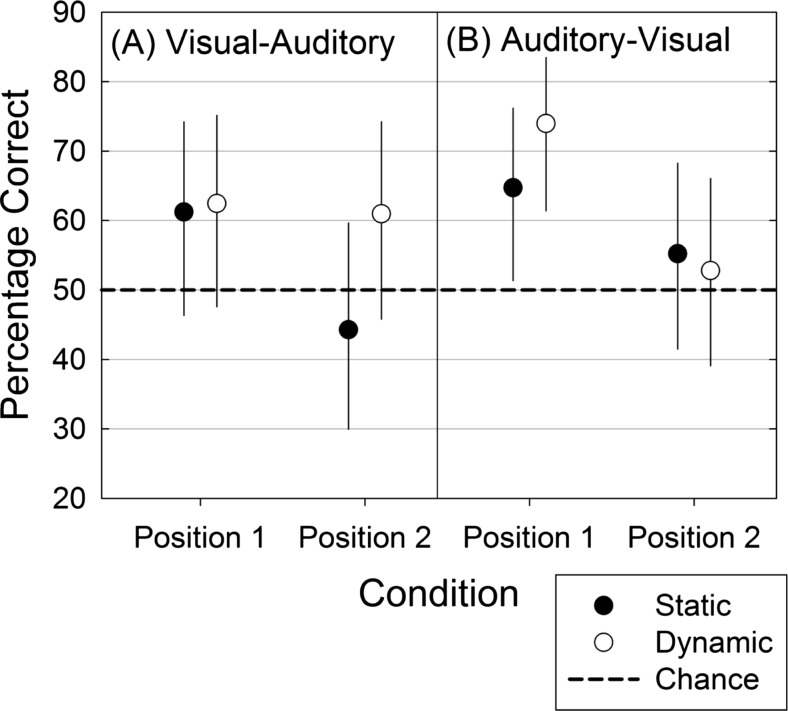
Face–voice matching accuracy on visual–auditory (panel A) and auditory–visual (panel B) trials for sequentially presented faces and voices in a two-alternative forced choice task. Error bars show 95 % confidence intervals for the condition means

The main effect of position was significant, along with the three-way interaction between position, order, and facial stimulus type. Figure 2 aids interpretation of the effects and interaction, showing means and 95 % confidence intervals for the percentage accuracies in each condition of the factorial design. The confidence intervals were obtained by simulating the posterior distributions of the cell means in R (arm package, version 1.6; Gelman & Su, 2013).

Overall, matching performance was significantly above the chance (50 %) level, *M =* 59.7 %, 95 % CI [50.8, 68.0]. However, the confidence intervals for percentage accuracy in the static (*M =* 57.6 %, 95 % CI [47.5, 67.1]) and dynamic (*M =* 63.7 %, 95 % CI [53.8, 72.5]) conditions show that only performance on dynamic facial stimulus trials was significantly above chance level. Figure 2 shows the main effect of position, with accuracy levels being consistently higher when the correct, matching other-modality stimulus was presented in Position 1 than when it was presented in Position 2. The results from the V–A condition are shown in panel A, whereas results from the A–V condition appear in panel B. The basis of the three-way interaction appears to relate to performance when the matching other-modality stimulus appears in Position 2 in the V–A condition. In that condition there was no position effect in the dynamic facial stimulus condition. However, as with any factorial design testing multiple effects, it would be imprudent to overinterpret a single nonpredicted interaction that is only just statistically significant (*p =* .037).

### Discussion

Using the standard crossmodal matching task (Lachs, 1999) employed in audiovisual speech perception research, in Experiment 1 we observed above-chance dynamic face–voice matching, but chance-level static face–voice matching. Although there was no significant difference between static and dynamic face–voice matching accuracy, and although static face–voice matching was close to being above chance level, this pattern of results appears to support the conclusion that the source identity information shared by dynamic articulating faces and voices explains accurate face–voice matching. The results are consistent with those of two previous studies (Kamachi et al. 2003; Lachs & Pisoni, 2004a), but are in conflict with Mavica and Barenholtz (2013, Exp. 2), who observed above-chance-level static face–voice matching using this procedure.

The presence of a position effect in Experiment 1 additionally suggests that memory load might be hindering performance, especially in the static facial stimulus condition. Matching was more accurate when the matching face and voice were presented close together in time (Position 1) than when the matching other-modality stimulus was further away, in Position 2. In line with research suggesting that memory is better for dynamic than for static faces (Christie & Bruce, 1998; Knappmeyer et al. 2003), the position effect did not manifest in the dynamic facial stimulus, V–A condition. This is the condition in which the face (Stimulus 1) would need to be held in memory for the longest time.

## Experiment 2

In order to clarify the effect of procedural differences across previous studies, in Experiment 2 we used a modified version of the presentation procedure from Experiment 1. Experiment 2 presented two different face–voice combinations. This time, the face and voice in each combination were presented simultaneously, instead of sequentially. By reducing the memory load, we hypothesized that matching accuracy might be higher when faces and voices were presented simultaneously, and above chance for static face–voice matching.

### Method

The methods for Experiment 2 were identical to those of Experiment 1, with the exceptions outlined below.

#### Participants

Seven male and 33 female adult participants (*N =* 40) took part in the experiment, with an age range of 18 to 33 years (*M =* 21.38, *SD =* 3.57). None of the participants had taken part in Experiment 1.

#### Procedure

**Fig. 3:**
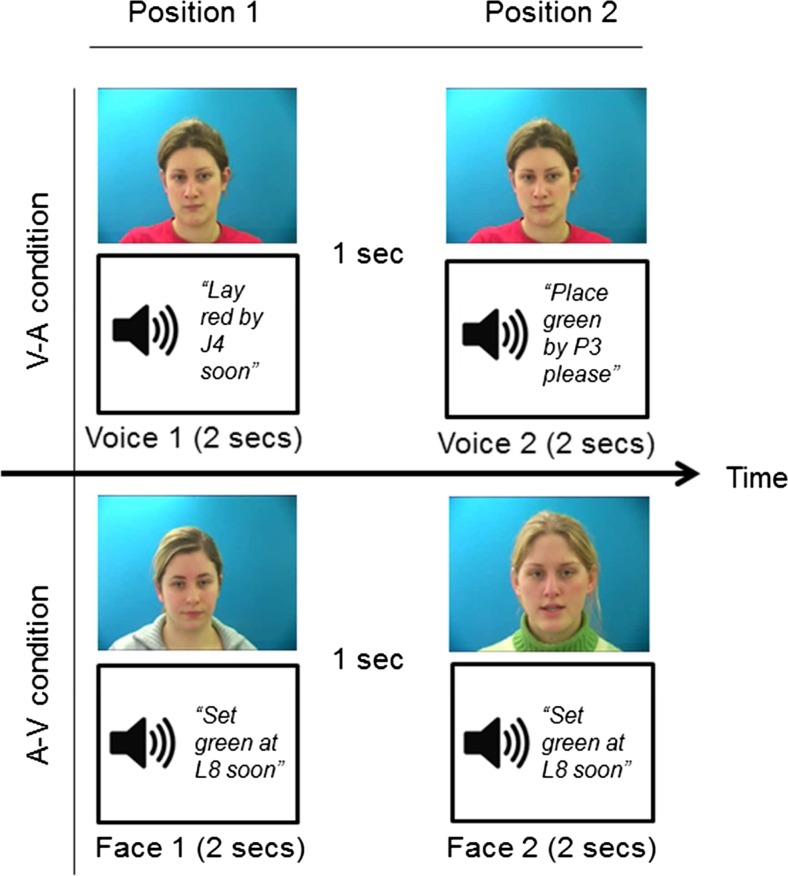
Procedure used in Experiment 3

The procedure used in Experiment 2 is illustrated in Fig. 3. Participants in the V–A condition saw a face accompanied by a recording of a voice. The text “Voice 1” was visible underneath the face. After a 1-s gap, they saw the same face accompanied by a different voice, and the text “Voice 2” appeared beneath the face. In the A–V condition, participants heard a voice accompanied by a face, then a 1-s intervening gap, before hearing the same voice accompanied by a different face. The text “Face 1” and “Face 2” appeared below the first and second combinations, respectively. Participants had to decide which combination was correct by pressing “1” for face–voice Combination 1, or “2” for face–voice Combination 2.

### Results

Face–voice matching accuracy was analyzed using the same method as in Experiment 1. Table 2 shows the likelihood chi-square statistic (*G*^2^) and *p* value associated with dropping each effect in turn from the appropriate model. The coefficients and standard error (on a log odds scale) for each effect in the full three-way interaction model are also reported in Table 2. We observed a similar pattern of *SD*s for the random effects. In the three-way model, the estimated *SD* of the first-stimulus random effect was .778; for the voice foils it was .324; and for the face foils it was .103. The estimated *SD* for the participant effect was .007.

**Table 2:** Parameter estimates (*b*) and likelihood ratio tests for the 2 × 2 × 2 factorial analysis, Experiment 2: Simultaneous face–voice presentation

*Source*	*df*	*b*	*SE*	*G* ^*2*^	*p*
Intercept	1	0.266	0.365	–	–
Position	1	0.550	0.462	17.40	<.001
Order	1	0.755	0.431	<0.01	.952
Facial Stimulus Type	1	0.314	0.391	0.37	.545
Position × Order	1	1.402	0.653	1.95	.162
Position × Facial Stimulus Type	1	0.140	0.568	1.09	.295
Order × Facial Stimulus Type	1	0.771	0.549	0.37	.544
Position × Order × Facial Stimulus Type	1	1.121	0.804	1.90	.169

**Fig. 4:**
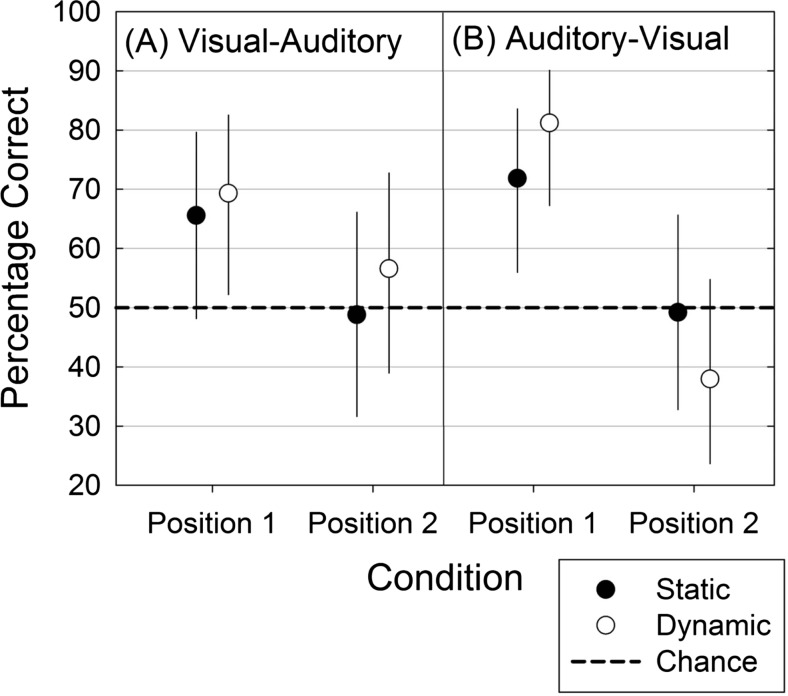
Face–voice matching accuracy on visual–auditory (panel A) and auditory–visual (panel B) trials for simultaneously presented faces and voices in a two-alternative forced choice task. Error bars show 95 % confidence intervals for the condition means.

Only the main effect of position was significant. Figure 4 aids interpretation of this main effect, showing the means and 95 % confidence intervals for accuracy in each of the eight conditions, obtained using the arm package (version 1.6; Gelman & Su, 2013).

As in Experiment 1, the overall matching performance was significantly above chance (50 %) level, *M =* 60.9 %, 95 % CI [50.4, 70.5]. Dynamic facial stimulus trials overall were significantly above chance (*M =* 62.5 %, 95 % CI [50.1, 73.6]), but static facial stimulus trials were not (*M =* 59.8 %, 95 % CI [47.2, 71.2]). As is clear from Fig. 4, the main effect of position exhibits the same pattern as in Experiment 1, with accuracy levels being consistently higher when the correct face–voice combination is presented in Position 1. There is, however, no three-way interaction.

### Discussion

Overall, the pattern of results observed in Experiment 2 is largely similar to that observed in Experiment 1, when all of the stimuli were presented sequentially. The participants in Experiment 2 exhibited a bias toward selecting the first face–voice combination they encountered. As the position effect was observed in both experiments, this may be attributable to the nature of the 2AFC task: When alternatives are presented sequentially, the first alternative is disproportionately favored. Indeed, as we noted in the introduction, other studies have shown widespread evidence of position biases using 2AFC procedures (García-Pérez & Alcalá-Quintana, 2011; Yeshurun et al. 2008). No three-way interaction was detected in Experiment 2. Thus, although the position effect may vary in strength depending on stimulus type and order, the two experiments presented here do not provide compelling evidence for this conclusion.

## Experiment 3

The results from Experiment 2 showed that simultaneously presenting faces and voices does not improve static face–voice matching. This was contrary to what we expected; it seems that the pattern of results from Experiment 1 was not attributable to increased memory load impairing the comparison of the first stimulus to the matching other-modality stimulus in Position 2. In Experiment 3, we aimed to test whether chance-level static face–voice matching could be attributable to the sequential presentation of alternatives in a 2AFC task. Evidence from the forensic eyewitness literature suggests that simultaneously presenting faces in a lineup array produces a different pattern of results than when faces are presented sequentially (Clark, Howell, & Davey, 2008; Ebbesen & Flowe, 2002; Steblay, Dysart, & Wells, 2011). This possibly occurs because of the differential use of relative and absolute judgments (Kneller, Memon, & Stevenage, 2001). Relative judgments (G. L. Wells, 1984) are employed when choosing the best option from simultaneously presented alternatives, whereas the sequential presentation of alternatives encourages absolute judgments because of the difficulty of making comparisons (G. L. Wells et al. 1998).

Some previous experiments finding above-chance accuracy with static stimuli have used a procedure in which the test alternatives were presented simultaneously, and can therefore be compared more easily (Krauss et al., 2002; Mavica & Barenholtz, 2013, Exp. 1). Experiment 3 tested whether static face–voice matching is above chance level when the alternatives in a 2AFC task are presented simultaneously. Because of the nature of this procedure, and the difficulty of presenting voices simultaneously at test, Experiment 3 only included an A–V condition. Although we did not expect a spatial position effect to manifest when the two face alternatives were presented simultaneously, we were unsure (in face of the contradictory previous research) whether this procedure would elicit above-chance static face–voice matching.

### Methods

#### Design

For Experiment 3, we employed a within-subjects design, with one factor: Spatial Position (left *=* Position 1, or right *=* Position 2). The dependent variable was matching accuracy.

#### Participants

Eight male and 22 female adult participants (*N =* 30) took part, with an age range of 18 to 44 years (*M =* 20.70, *SD =* 5.20). The participants were recruited in the same way as in Experiments 1 and 2, although none had taken part in previous experiments. All participants reported having normal or corrected vision and hearing.

#### Apparatus and materials

The software and equipment used in Experiments 1 and 2 were also used in Experiment 3. The voice stimuli and static facial stimuli were also the same as in the previous experiments. In the absence of a between-subjects manipulation, only four versions of Experiment 3 were constructed, all of which featured different combinations of stimuli. Each version featured one block of 18 trials, in which a voice was followed by the presentation of two faces. The same-identity face was always present at test, with its spatial position (left *=* Position 1 or right *=* Position 2) being randomly and equally varied. Each voice was only heard once in each version. Each of the stimulus faces appeared twice, but only once as the correct, matching stimulus. This was in keeping with the procedure of Krauss et al. (2002), who also reused the visual stimuli as foils within blocks.

#### Procedure

**Fig. 5:**
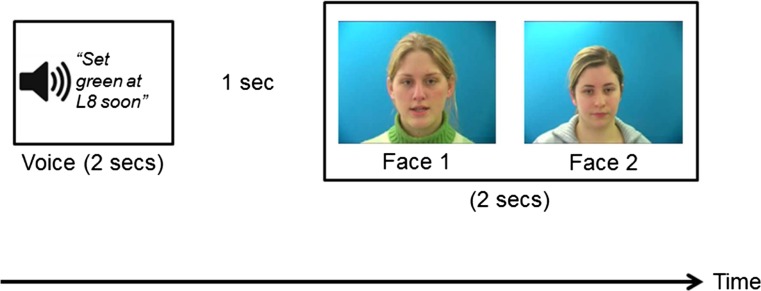
Procedure used in Experiment 3

The participants were randomly allocated to one of the four experimental versions using an online research randomizer (Urbaniak & Plous, 2013). As is illustrated in Fig. 5, participants heard a voice for 2 s. After a 1-s gap, they saw two images of faces presented side by side. The text “Face 1” was visible underneath the face on the left, and the text “Face 2” appeared underneath the face on the right. This screen was visible for 2 s. Participants were then instructed to decide which face matched the voice they had heard, indicating their answer by pressing “1” for “Face 1” or “2” for “Face 2.”

### Results

Face–voice matching accuracy was analyzed using the same method as in Experiments 1 and 2. Since there was only one within-subjects factor, we only report the likelihood chi-square statistic (*G*^2^) and *p* value associated with dropping the main effect from the null model. The coefficients and standard error (on a log odds scale) for the effect of spatial position in the main effect model are reported in Table 3. In the main effect model, the estimated *SD* of the voice random effect was .487, and that for the face foil was .0002. The estimated *SD* for the participant effect was less than .0001.

**Table 3:** Parameter estimates (*b*) and likelihood ratio tests for the analysis, Experiment 3: Simultaneously presented alternatives

*Source*	*df*	*b*	*SE*	*G* ^*2*^	*p*
Intercept	1	0.446	0.147	–	–
Spatial Position	1	0.199	0.203	0.98	.329

The main effect of spatial position was nonsignificant, and the overall matching accuracy with simultaneously presented static facial stimuli was above chance level (50 %), *M =* 61.0 %, 95 % CI [54.1, 67.6].

### Discussion

The results indicate that when test alternatives are presented simultaneously, static face–voice matching is above chance level. In keeping with the previous results (Mavica & Barenholtz, 2013; Smith et al., 2015), this confirms that static face–voice matching is possible. The results also replicate the findings of Krauss et al. (2002), but using headshots rather than full-length images. When we consider these alongside the results presented in Experiments 1 and 2, it appears that static face–voice matching performance is sensitive to procedure, thus offering one possible explanation for the contradictions between previous studies.

Experiments 1 and 2 showed that there is a temporal position bias when test options are presented sequentially. However, Experiment 3 suggests that there is no corresponding spatial position bias; when the test options are presented simultaneously, the position bias is negligible.

## General discussion

In an attempt to resolve the discrepancies across previous face–voice matching studies, the three experiments presented here tested whether crossmodal source identity information is exclusively dependent on encoding visual articulatory patterns, or whether static faces and voices offer sufficient concordant information to facilitate above-chance performance. Taken together, the results are consistent with the conclusion that, although articulatory movement might be important in facilitating face–voice matching (Exps. 1 and 2), it is also possible to match static faces and voices when a 2AFC procedure facilitates comparisons between the alternatives (Exp. 3). Therefore, it seems that the procedural differences between previous studies offer a possible explanation for the discrepant results in the literature. Furthermore, as was shown by the variance associated with the stimuli in the multilevel modeling analysis, people vary in the extent to which they look and sound similar. This offers a complementary explanation for the contradictions in previous studies, because results may be highly dependent on the particular stimuli used.

### Static versus dynamic face–voice matching

In Experiments 1 and 2, we presented the test alternatives in the 2AFC task sequentially. The results replicated those of audiovisual speech perception studies, showing that although dynamic faces and voices can be matched at a level significantly above chance, static faces and voices cannot (Kamachi et al., 2003; Lachs & Pisoni, 2004a). However, static face–voice matching was very close to being above chance level, and there was no significant difference between the facial stimulus conditions. These results hint at the existence of a trend toward accurate static face–voice matching across all three experiments. As was shown by the results of Experiment 3, and in keeping with the hypothesis that static faces and voices also offer concordant source identity information (Feinberg et al., 2005; Krauss et al., 2002; Mavica & Barenholtz, 2013; Saxton, Caryl, & Roberts, 2006; Smith et al., 2015), when the alternatives were presented simultaneously, performance was significantly above chance. The overall results are therefore not consistent with the conclusion that dynamic articulatory movement is exclusively responsible for explaining crossmodal matching (e.g., Kamachi et al., 2003; Lachs & Pisoni, 2004a), although they do not rule out the audiovisual speech perception argument that visual articulatory movement shares source identity information with voices (Kamachi et al., 2003; Lachs & Pisoni, 2004a, b; Rosenblum et al., 2006).

The lack of a statistical difference between static and dynamic face–voice matching in Experiments 1 and 2 corresponds with the results of previous findings using a same–different procedure (Smith et al., 2015). This warns against overstating the importance of visual articulatory movement in accounting for crossmodal matching accuracy. That said, the lack of an effect of facial stimulus type is not necessarily at odds with the results of studies that have detected accurate face–voice matching when movement was isolated using point-light displays and static information was unavailable (Lachs & Pisoni, 2004b; Rosenblum et al., 2006). Dynamic point-light displays could offer sufficient information to inform accurate face–voice matching, independently of the structural information available in static images.

### Procedural differences

On both static and dynamic facial stimulus trials, we observed a uniform position effect in Experiment 2 when the memory load was reduced. This finding suggests that the discrepant pattern of results across previous studies is not a consequence of differential memory effects for static and dynamic faces. Rather, our findings are more consistent with the conclusion that the position effect is attributable to the nature of the 2AFC task (García-Pérez & Alcalá-Quintana, 2011; Yeshurun et al., 2008) when the two test alternatives are presented sequentially. In keeping with this argument, the position effect disappeared when the static alternatives were presented simultaneously, in Experiment 3.

Alternatively, the position effect might have manifested because faces and voices are most commonly perceived simultaneously during social interactions. Therefore, participants may have exhibited a bias to accept a face and voice presented in relative temporal proximity (Exp. 1) or the combination presented first (Exp. 2) as coming from the same person. This explanation would disproportionately support matching accuracy when the matching other-modality stimulus appears in Position 1, in line with the position bias observed in both Experiment 1 and 2.

In comparing the results of Experiments 1 and 2 to those of Experiment 3, it appears that static face–voice matching is sensitive to the procedure employed. The similarity of the results across Experiments 1 (sequential face–voice presentation) and 2 (simultaneous face–voice presentation) suggest that the contradictions between previous studies are not attributable to superior performance when faces and voices are presented simultaneously. This may occur because the more critical comparison to make in facilitating matching accuracy is between alternatives, rather than between the face and the voice. When the two alternatives are presented simultaneously, as in Experiment 3, the key comparison, a relative judgment (Wells, 1984), is easier to make.

At this point, it should be noted that in previous face–voice matching experiments using a crossmodal matching procedure, a standard interstimulus interval of 500 ms has been used (e.g., Lachs & Pisoni, 2004a, b; Mavica & Barenholtz, 2013), which is half as long as the interval featured in the experiments we report. With 1-s intervals in Experiment 1, we observed chance-level static face–voice matching when the stimuli were presented sequentially. Using 500-ms intervals, Mavica and Barenholtz (2013, Exp. 2) observed above-chance-level matching accuracy. It is necessary to consider the possible impact of this methodological dissimilarity. It could be argued that a longer interval might increase the load on auditory and visual sensory memory, making the task more difficult. The results that we report support the argument that sensory memory pressures do not account for the chance-level static facial stimulus results in Experiment 1. Experiment 2, in which faces and voices were presented simultaneously, was designed to alleviate memory load, and the results were very similar to those of Experiment 1: Static face–voice matching was still at chance level.

### Variability associated with the stimuli

An explanation based on procedural differences does not accommodate all of the results in the previous literature. Mavica and Barenholtz (2013) observed above-chance static face–voice matching using sequential presentation of alternatives in the A–V condition of the standard crossmodal matching task (Lachs, 1999). Alongside procedural differences, our set of three experiments also highlights the importance of stimulus variability in providing an additional, but complementary, explanation for the contradictions between previous studies. Other studies have used varying numbers of face–voice pairs when testing crossmodal matching. For example, Lachs and Pisoni (2004a) used eight pairs of stimuli, but Kamachi et al. (2003) used 40. Our multilevel modeling analysis revealed that some people look and sound more similar than others; relatively high levels of variance associated with the stimuli were observed for the 18 face–voice pairs used here, and in all three experiments, the overall variance associated with stimuli was far greater than that associated with participants. Consistent with this, Mavica and Barenholtz reported that for their stimuli, levels of matching accuracy varied widely, between 35 % and 70 %, across 64 face–voice pairs. Overall, Mavica and Barenholtz’s stimulus pairings of voices and static faces may have been easier to match than the pairings featured in our study, or than those featured in previous studies (Kamachi et al., 2003; Lachs & Pisoni, 2004a).

A key strength of the present research is our use of multilevel modeling. Although Mavica and Barenholtz (2013) ran a power analysis indicating that the discrepancies between previous studies were not due to lack of statistical power, simultaneously accounting for variance associated with stimuli and participants is a problem that can only be appropriately dealt with by running a multilevel model (Baguley, 2012; Judd et al., 2012). This statistical approach allows generalizations to be made across both stimuli and participants, and is generally more conservative than traditional analyses such as ANOVA, which aggregate over one or the other variable. However, multilevel modeling has not been previously used when investigating face–voice matching, reducing confidence in the generality of the findings in this field.

### No order effects in 2AFC tasks

In line with other studies (Kamachi et al., 2003, forward and backward conditions; Lachs & Pisoni, 2004a; Lander et al., 2007), neither Experiment 1 nor 2 showed an effect of order. Although some asymmetries were found between V–A and A–V conditions in Smith et al.’s (2015) same–different procedure, the results suggested that these asymmetries were owing to a response bias on A–V trials. We would not expect such an effect to manifest in a 2AFC paradigm, which tests sensitivity rather than response bias.

### Conclusion

The results of the three experiments reported here suggest that source identity is shared by dynamic articulating faces and voices, as well as by static faces and voices. Our findings help resolve previous uncertainty about whether static face–voice matching is possible, presenting two complementary explanations for the apparent contradictions. The data suggest that static face–voice matching is more likely to be above chance level when the alternatives in a 2AFC task are presented simultaneously. In addition, the variance associated with stimuli indicates that some people look and sound more similar than others, an issue that has not been properly accounted for by the analyses undertaken in previous research, but that helps explain why the static face–voice matching performance across previous studies might be inconsistent. Our results therefore support the conclusion that dynamic visual information about articulatory patterns facilitates accuracy (Kamachi et al., 2003; Lachs & Pisoni, 2004a, b; Lander et al., 2007; Rosenblum et al., 2006), but that it alone cannot explain the existence of shared source identity information with voices. Crossmodal source identity information is available in both static and dynamic faces.
